# A Unique Case of Non-paraneoplastic Lambert-Eaton Myasthenic Syndrome Treated With Subcutaneous Immunoglobulin: A Case Report and Review of Literature

**DOI:** 10.7759/cureus.60773

**Published:** 2024-05-21

**Authors:** Sojung K Park, Melanie G Taylor

**Affiliations:** 1 Neurology, Trinity Health Grand Rapids, Grand Rapids, USA; 2 Neurology, Michigan State University College of Human Medicine, Grand Rapids, USA

**Keywords:** neuromuscular diseases, electromyography (emg), subcutaneous immunoglobulin, intravenous immunoglobulin (ivig), lambert-eaton myasthenic syndrome

## Abstract

Lambert-Eaton myasthenic syndrome (LEMS) is an autoimmune neuromuscular disorder caused by pathogenic autoantibodies directed against voltage-gated calcium channels present on the presynaptic nerve terminal. For LEMS patients refractory to initial symptomatic treatment with amifampridine, immunomodulatory therapy with intravenous immunoglobulin (IVIG) is often utilized. However, in the authors' review of literature, the utility of subcutaneous immunoglobulin (SCIG) in the treatment of LEMS has been scarcely reported. Here, we present a unique case of non-paraneoplastic LEMS managed with SCIG with excellent clinical response and improvement on electromyography. SCIG therapy may be a reasonable alternative for patients with LEMS who do not tolerate the intravenous formulation.

## Introduction

Lambert-Eaton myasthenic syndrome (LEMS) is an autoimmune neuromuscular disorder caused by pathogenic autoantibodies directed against voltage-gated calcium channels (VGCC) present on the presynaptic nerve terminal [1]. LEMS is a rare entity with a reported estimated incidence of 0.48 per million [2]. Approximately 50-60% of LEMS cases are paraneoplastic, usually associated with small-cell lung carcinoma, which also expresses functional VGCC [1]. Paraneoplastic LEMS (P-LEMS) occurs mostly in men with a median age of onset of 60 years [3]. The remainder of LEMS cases, non-paraneoplastic LEMS (NP-LEMS), are not associated with malignancy and tend to affect women slightly more (52%) than males with a peak age of onset around 35 years and a larger, second peak at age 60 years [3]. 

The classic clinical triad of LEMS consists of proximal muscle weakness, areflexia, and autonomic dysfunction [4]. There are many therapeutic agents available for LEMS management including amifampridine (also known as 3,4-diaminopyridine), pyridostigmine, prednisolone, azathioprine, immunoglobulin, and plasma exchange [5,6]. There has been recent evidence that patients with NP-LEMS have normal survival and that most patients with LEMS achieve a stable disease course and are able to remain or become independent for activities of daily living [7]. There is a crucial need to recognize symptoms of LEMS and to diagnose patients promptly, thereby facilitating treatment and improving quality of life.

## Case presentation

A 27-year-old female with a history of postpartum depression developed weakness affecting the proximal bilateral lower extremities. Her weakness was worse in the morning and improved throughout the day. Symptoms worsened over several years, and she was diagnosed with fibromyalgia and chronic fatigue syndrome. 

**Figure 1:**
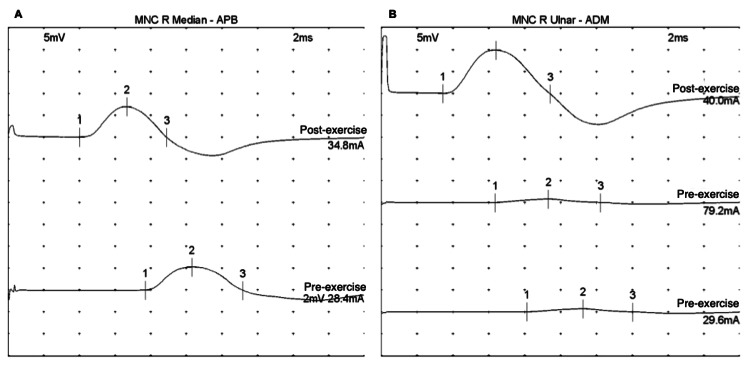
Initial EMG prior to treatment (A) EMG of the right median nerve at the APB muscle. The pre-exercise (bottom row) nerve conduction study demonstrates reduced CMAP. After 10 seconds of maximal voluntary contraction, post-exercise facilitation is noted (top row). (B) EMG of the right ulnar nerve at the ADM muscle. The pre-exercise studies (middle and bottom rows) demonstrate markedly reduced CMAPs. After 10 seconds of maximal voluntary contraction, post-exercise facilitation is noted (top row). EMG: electromyography; CMAP: compound motor action potential; APB: abductor pollicis brevis; ADM: abductor digiti minimi

The patient was eventually assessed in the neuromuscular clinic. Her physical exam revealed bilateral proximal lower extremity weakness and diffuse hyporeflexia, which improved with brief exercise. Workup demonstrated positive for P/Q VGCC antibodies with a titer of 0.78 nmol/L (normal range 0.02 nmol/L or less). Electromyography (EMG) showed post-exercise compound motor action potential (CMAP) facilitation in the right median and ulnar nerves (Figure 1). This strongly supported the diagnosis of LEMS.

There were 233.3% and 1150.0% increases from pre- to post-exercise amplitudes in median and ulnar nerves, respectively (Table 1).

**Table 1:** Data from median and ulnar motor nerve conduction studies prior to treatment *Change in amplitude (%)=(post-exercise amplitude−pre-exercise amplitude)/pre-exercise amplitude×100% APB: abductor pollicis brevis; ADM: abductor digiti minimi

Nerve/sites	Latency (ms)	Amplitude (mV)	Segments	Distance (mm)	Temp. (°C)
R median - APB					
Pre-exercise	4.0	2.1	Wrist - APB	80	32
Post-exercise	4.0	7.0	Wrist - APB	80	32
Change in amplitude (%)*		233.3			
R ulnar - ADM					
Pre-exercise	3.4	0.8	Wrist - ADM	80	32.2
Post-exercise	3.4	10.0	Wrist - ADM	80	32.5
Change in amplitude (%)*		1150.0			

Computed tomography (CT) of the chest, abdomen, and pelvis did not reveal evidence of malignancy. The erythrocyte sedimentation rate (ESR) was 31 mm/hr (reference value <20). The C-reactive protein, creatine kinase, and complement C3 and C4 values were within the normal range. Striated muscle antibodies and acetylcholine receptor binding antibodies were negative. Complete blood count, comprehensive metabolic panel, and thyroid-stimulating hormone levels were all within normal limits. Magnetic resonance imaging (MRI) of the brain was unremarkable. 

The patient was started on amifampridine which provided some symptom improvement. She was treated with intravenous immunoglobulin (IVIG) for further management, but she developed intolerable headache and myalgias. Subsequently, she was started on subcutaneous immunoglobulin (SCIG) 400 mg/kg/week. This was well tolerated and resulted in excellent clinical response. She reported significant improvement in her weakness and was able to resume her occupation as a teacher. Her muscle strength and reflexes improved on repeat neurologic examination a year after the initiation of SCIG (Table 2).

**Table 2:** Motor strength and reflexes before and after SCIG treatment SCIG: subcutaneous Immunoglobulin

	Before SCIG		One year after starting SCIG	
Motor strength	Right	Left	Right	Left
Shoulder flexion	5/5	5/5	5/5	5/5
Shoulder abduction	5/5	5/5	5/5	5/5
Elbow flexion	5/5	5/5	5/5	5/5
Elbow extension	5/5	5/5	5/5	5/5
Hand grip	5/5	5/5	5/5	5/5
Hip flexion	3/5	4/5	5/5	5/5
Hip extension	3/5	4/5	5/5	5/5
Hip abduction	4/5	4/5	5/5	5/5
Hip adduction	4/5	4/5	5/5	5/5
Knee flexion	4/5	5/5	5/5	5/5
Knee extension	4/5	4/5	5/5	5/5
Ankle dorsiflexion	5/5	5/5	5/5	5/5
Ankle plantarflexion	5/5	5/5	5/5	5/5
Ankle inversion	5/5	5/5	5/5	5/5
Ankle eversion	5/5	5/5	5/5	5/5
Reflexes				
Biceps	1+	1+	2+	2+
Brachioradialis	1+	1+	2+	2+
Triceps	2+	2+	2+	2+
Patellar	1+	1+	2+	2+
Achilles	1+	Trace	2+	1+

**Figure 2:**
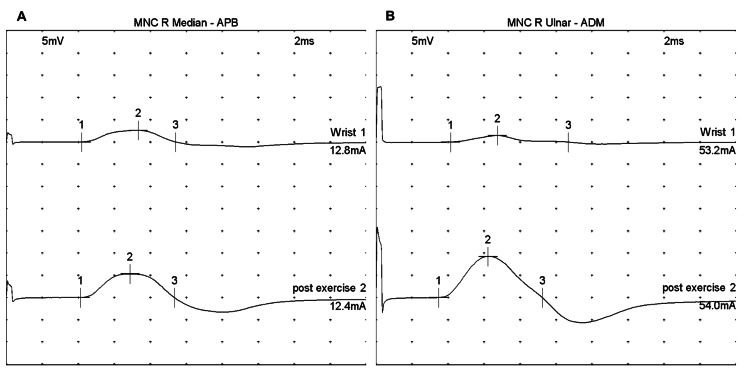
Repeat EMG one year after SCIG treatment (A) EMG of the right median nerve at the APB muscle. The pre-exercise (top row, labeled "Wrist 1") nerve conduction study demonstrates reduced CMAP. After 10 seconds of maximal voluntary contraction, post-exercise facilitation is noted (bottom row, labeled "post exercise 2"). (B) EMG of the right ulnar nerve at the ADM muscle. The pre-exercise study (top row, labeled "Wrist 2") demonstrates markedly reduced CMAPs. After 10 seconds of maximal voluntary contraction, post-exercise facilitation is noted (bottom row, labeled "post exercise 2"). EMG: electromyography; SCIG: subcutaneous immunoglobulin; CMAP: compound motor action potential; APB: abductor pollicis brevis; ADM: abductor digiti minimi

Repeat EMG after one year of being treated with SCIG was performed (Figure 2), which showed persistent facilitation consistent with LEMS. There was an improvement in the degree of facilitation compared to the initial study with 103.8% and 520.0% increases from pre- to post-exercise amplitudes in median and ulnar nerves, respectively (Table 3).

**Table 3:** Data from median and ulnar motor nerve conduction studies one year after treatment initiation *Change in amplitude (%)=(post-exercise amplitude−pre-exercise amplitude)/pre-exercise amplitude×100% APB: abductor pollicis brevis; ADM: abductor digiti minimi

Nerve/sites	Latency (ms)	Amplitude (mV)	Segments	Distance (mm)	Temp. (°C)
R Median - APB					
Pre-exercise	4.1	2.6	Wrist - APB	80	32
Post-exercise	4.2	5.3	Wrist - APB	80	32
Change in amplitude (%)*		103.8			
R Ulnar - ADM					
Pre-exercise	3.5	1.5	Wrist - ADM	80	32
Post-exercise	4.1	9.3	Wrist - ADM	80	32
Change in amplitude (%)*		520.0			

The patient is being managed with lifestyle modifications for orthostatic hypotension which is likely related to autonomic nervous system dysfunction related to LEMS. She was also diagnosed with Sjögren's syndrome and spondyloarthritis by rheumatology with serology demonstrating positive anti-SS-A (Ro), negative anti-SS-B (La) antibody, and positive HLA B27 typing. This highlighted her predilection for autoimmune disorders, which are associated with NP-LEMS.

## Discussion

LEMS is named after neurologists Lambert and Eaton who reported six patients with progressive muscle weakness, hyporeflexia, lung carcinoma, and a distinct pattern found on electrophysiology with repetitive nerve stimulation in 1956 [8]. Since the initial reports of cases, the immunopathophysiology of the disease has been extensively studied. The distinct weakness of striated skeletal muscles is due to the pathogenic autoantibodies to presynaptic VGCCs in the membrane of the motor nerve terminal, impairing the release of acetylcholine [8]. The autonomic dysfunction observed in LEMS is postulated to be related to the same mechanism involving VGCC antibodies. These antibodies impair neurotransmitter release from parasympathetic and sympathetic neurons via the downregulation of one or more subtypes of VGCCs [9]. Autonomic dysfunction symptoms may include orthostatic hypotension, keratoconjunctivitis sicca, ptosis, constipation, anhidrosis, and erectile dysfunction.

LEMS is frequently misdiagnosed. In one study, 58% of all cases were initially diagnosed incorrectly prior to establishing LEMS [10]. Our patient was misdiagnosed with fibromyalgia prior to evaluation in the neuromuscular clinic. A high degree of clinical suspicion is necessary to make the diagnosis. The diagnosis of LEMS should be raised in the classical triad of proximal muscle weakness, hyporeflexia, and autonomic dysfunction [4]. This should trigger further workup including laboratory and electrodiagnostic studies. Antibodies against presynaptic membrane P/Q-type VGCCs are highly sensitive, present in approximately 90% of LEMS patients [3]. Electrophysiological findings will classically demonstrate reduced CMAPS, significant decrements in the response to low-frequency (2-5 Hz) repetitive nerve stimulation, and increment responses after brief (10-30 seconds) maximal voluntary contraction or with high-frequency (20-50 Hz) stimulation [4]. 

In older patients with a history of tobacco use, a workup for underlying malignancy should be performed [7]. In a younger patient population without a history of smoking, the diagnosis is more likely to be associated with a general autoimmune state. NP-LEMS is associated with an increased susceptibility to other autoimmune diseases [4]. Our patient described above was found to have Sjögren's and non-radiographic axial spondyloarthritis in addition to LEMS, highly suggestive of her predisposition for autoimmune conditions in general. 

The mainstay of therapy for symptomatic patients with weakness includes amifampridine, a potassium channel blocker which prolongs the presynaptic nerve terminal membrane depolarization and enhances the influx of calcium, thereby improving the release of acetylcholine [11]. Pyridostigmine, an acetylcholinesterase inhibitor, may also be considered although it is often not as effective as when used for myasthenia gravis (MG) [12]. For patients refractory to initial symptomatic treatment with 3,4-diaminopyridine, immunomodulatory therapy with IVIG is considered [13,14]. 

In our review of literature, the utility of SCIG in the treatment of LEMS has been scarcely reported. However, it has been well described in the literature that the subcutaneous administration is associated with a lower incidence of adverse effects compared to IVIG, perhaps related to the slower absorption and lower peak serum levels [15]. It was effective and well tolerated in our patient with NP-LEMS. SCIG may be a reasonable alternative for patients with LEMS who do not tolerate the intravenous route. 

Plasma exchange can be employed for LEMS as well, and its effect is comparable to IVIG although not as often used due to technical difficulties and slightly higher rates of complications [3,16]. Immunotherapy with prednisolone and azathioprine has been well documented to have benefits in LEMS patients [6,17]. Mycophenolate mofetil, cyclosporine, and rituximab are sometimes utilized for LEMS treatment in refractory cases [16,18].

## Conclusions

The diagnosis of LEMS requires a high level of clinical suspicion, and prompt diagnosis is necessary to facilitate treatment. This report describes a unique case of NP-LEMS treated with SCIG. SCIG was well tolerated by our patient after experiencing intolerable adverse effects with IVIG. SCIG resulted in subjective symptom control with objective improvement in motor strength and reflexes. The subcutaneous administration may be a reasonable alternative form of immunomodulatory therapy for patients with LEMS who cannot otherwise be treated with IVIG. While the higher tolerability of SCIG compared to IVIG has been established, its use in LEMS patients is not well described in the literature to date. Further research is required to confirm the safety and efficacy of SCIG in LEMS patients.
